# A Green Synthesis of Controllable Shear-Assisted Catalytically Graphitized Biomass-Derived Carbon and Its Multi-Scale Reinforcement Mechanism in Natural Rubber

**DOI:** 10.3390/molecules30091936

**Published:** 2025-04-27

**Authors:** Xingxin Xu, Chengjun Li, Xu Lin, Defa Hou, Yunwu Zheng, Fulin Yang, Hao Sun, Can Liu

**Affiliations:** National Joint Engineering Research Center for Highly-Efficient Utilization Technology of Forestry Resources, Southwest Forestry University, Kunming 650224, China; 13505201631@163.com (X.X.); lcj202506@163.com (C.L.); linxu@swfu.edu.cn (X.L.); houdefa001@163.com (D.H.); zyw85114@163.com (Y.Z.); yangfulin0309@163.com (F.Y.)

**Keywords:** biochar, ball milling, natural rubber, mechanical properties

## Abstract

Carbon black (CB) serves as the most crucial reinforcing filler in natural rubber (NR) applications. However, conventional CB production relies on petroleum or coal resources, raising concerns about non-renewability and unsustainable resource consumption. Although biomass-derived carbon materials have been explored as alternatives for natural rubber reinforcement, their practical application remains constrained by inherent limitations such as large particle size and low graphitic structure, which compromise reinforcement efficiency. This study presents a novel walnut shell biochar (WSB) for natural rubber enhancement. The biochar was prepared via conventional pyrolysis and subsequently subjected to an environmentally friendly physical ball-milling process. This treatment effectively increased graphitized domains while enriching surface functional groups. Systematic investigations were conducted on the effects of ball-milling duration and biochar loading on rubber reinforcement performance. Results demonstrate that the biochar-reinforced vulcanizates achieved a 22% improvement in tensile strength compared to unfilled rubber. Notably, at 10 phr loading, the tensile strength of biochar-filled vulcanizates reached 98% of that achieved by CB(N330)-filled counterparts. The study further revealed that biochar incorporation effectively reduced hysteresis loss and enhanced elastic recovery in rubber composites. This work proposes a facile method to develop sustainable biochar-based reinforcing agents with significant potential for natural rubber applications.

## 1. Introduction

Natural rubber exhibits superior physical and mechanical properties, leading to its extensive applications in diverse fields ranging from daily life products to industrial production sectors. Raw natural rubber exhibits inherently limited mechanical properties, necessitating the incorporation of reinforcing agents followed by vulcanization to meet the performance requirements for practical applications [1,2,3]. The most widely utilized reinforcing agent in the contemporary rubber industry is carbon black [4]. It is generally recognized that the reinforcement performance of carbon black in rubber matrices is predominantly determined by its particle size, aggregate structure, and surface chemical properties. These characteristics enhance the dispersion stability of carbon black within the rubber matrix and strengthen the interfacial bonding between carbon black and the polymer network, thereby forming high-performance natural rubber composites [5,6]. Commercial carbon black is produced through the thermochemical conversion of non-renewable fossil fuels such as petroleum and coal. The production and utilization of carbon black have led to a range of adverse impacts on resources, the environment, and human health. These issues have prompted significant efforts to explore viable alternative substitutes for carbon black [7].

Biochar demonstrates comparable properties to carbon black, positioning it as a promising sustainable reinforcing filler alternative in natural rubber composites. As a reinforcing agent for natural rubber, biochar addresses critical environmental challenges including pollution mitigation, climate change adaptation, and fossil resource conservation, while concurrently reducing manufacturing costs and advancing circular economy principles. This innovation offers technical support for China’s green tire industry development and aligns with national “Dual Carbon” strategic objectives. Previous investigations have explored biochar applications in rubber performance enhancement [8,9,10], with notable examples including coconut shell-derived biochar [11], leaf-derived activated carbon [12], palm kernel shell biochar [13], and bamboo-based biochar [14]. While these advancements contribute to fossil fuel conservation and low-cost biobased filler production, their reinforcement efficacy remains inferior to high-structure commercial carbon black. China’s walnut cultivation yields 4.8 million metric tons annually (36% of global production in 2022), generating approximately 960,000 metric tons of walnut shell byproducts [15]. Comprising lignin (50.3%), cellulose (23.9%), and hemicellulose (22.4%) [16,17], walnut shells present an optimal feedstock for biochar production. We propose that walnut shell-derived biochar exhibits significant potential as a sustainable carbon black alternative for rubber reinforcement applications.

The appropriate small particle size and surface functional groups of biochar play a critical role in enhancing the mechanical properties of rubber composites, including tensile strength, toughness, and storage modulus [9,18]. However, biochar’s inherent limitations, such as large particle size and low surface activity, make it challenging to directly enhance the mechanical performance of rubber matrices, necessitating effective modification strategies. Current modification methods for biochar mainly include gas activation, chemical doping, and microbial attachment [19], yet these approaches often suffer from operational complexity, potential generation of hazardous byproducts, and high implementation costs. In contrast, ball milling has emerged as a simple, environmentally benign, and efficient technique capable of simultaneously achieving surface modification and uniform particle refinement [20,21,22]. In recent years, ball milling has been applied for biochar modification to enhance the performance of rubber materials. Beichen Xue et al. [23] demonstrated that ball milling not only effectively reduced the particle size of pyrolyzed rice husk but also modified its surface characteristics and porous structure. Similarly, Can Jiang et al. [24] employed ball milling to obtain finer lignin-derived biochar particles. 

**Figure 1:**
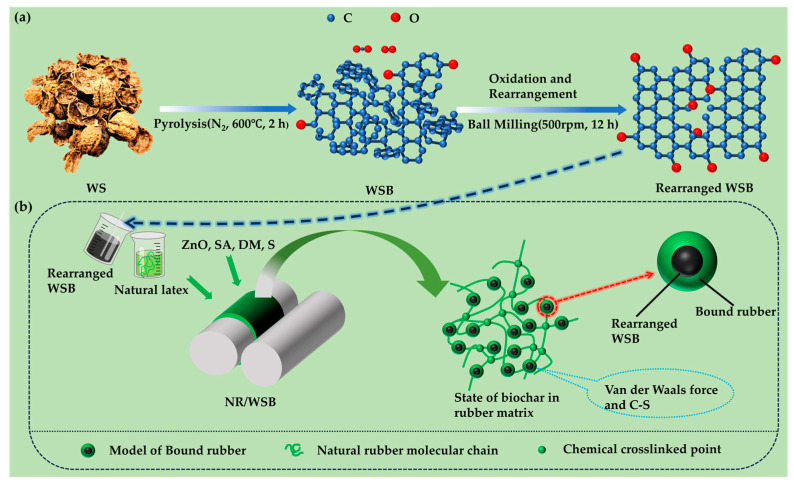
(**a**) Schematic diagram of structural transformation and composition evolution mechanism of WSB; (**b**) reinforcement mechanism of WSB in rubber matrix.

This study aims to develop a sustainable rubber reinforcement material based on agricultural waste. Herein, WSB was prepared from agricultural waste walnut shells and the effects of ball-milling duration on its physicochemical properties were systematically investigated. The study further examined how varying ball-milling durations of WSB and different WSB loadings influenced the vulcanization characteristics and mechanical properties of natural rubber composites (Figure 1a,b). Additionally, a comparative analysis was conducted to evaluate the reinforcement effects between WSB and commercial carbon black (N330) in natural rubber matrices. This work demonstrates the potential of walnut shell biochar as a substitute for carbon black in the rubber industry, providing scientific evidence for replacing petroleum-based carbon black with biochar and promoting the high-value utilization of agricultural waste in rubber applications.

## 2. Results

### 2.1. Physicochemical Characterization of Fillers

**Figure 2:**
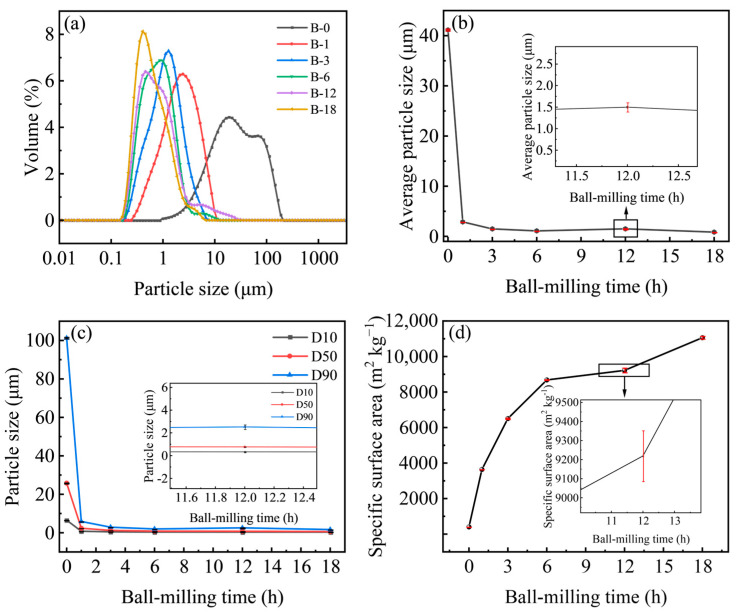
(**a**) The particle size distribution of the ball milled products; (**b**) the average particle sizes of the ball milled products; (**c**) results for D10, D50 and D90 (D10: Particles having a particle size less than this value account for 10% of the total volume, and so on for D50 and D90); (**d**) the specific surface area of the ball milled products.

The particle size of fillers is a critical factor influencing the mechanical properties of rubber. Figure 2 demonstrates the effectiveness of ball milling in reducing the particle size of WSB. The unmilled WSB exhibited a broad size distribution with particles exceeding 40 μm and reaching approximately 180 μm. With increasing ball-milling duration, the size distribution peak progressively shifted towards smaller dimensions, achieving 0.46 μm after 12 h and further decreasing to 0.41 μm after 18 h. Notably, although prolonged ball milling beyond 12 h resulted in higher bulk density peaks, no significant reduction in particle size was observed thereafter (Figure 2a). The particle size distribution parameters of WSB, including the median diameter (D50) and percentile diameters (D10 and D90), exhibited a progressive refinement trend. This phenomenon was attributed to the intensive mechanical impact and grinding effects generated during the continuous ball-milling process, which effectively reduced particle dimensions through sustained size-reduction mechanisms. At a ball-milling duration of 12 h, the average particle size and D90 of WSB exhibited slight increases. This phenomenon may be attributed to the generation of carbon-based radicals during mechanical processing, which introduces oxygen-containing functional groups on particle surfaces. These functional groups elevate surface energy, thereby promoting particle aggregation through electrostatic interactions, hydrogen bonding, and other intermolecular forces, ultimately forming loosely bound agglomerates [25,26,27]. With prolonged ball milling, these agglomerates underwent subsequent fragmentation into finer particles, as evidenced by the morphological evolution shown in Figure 2b,c. Concurrently, prolonged ball-milling duration progressively increases the specific surface area of WSB (Figure 2d).

**Figure 3:**
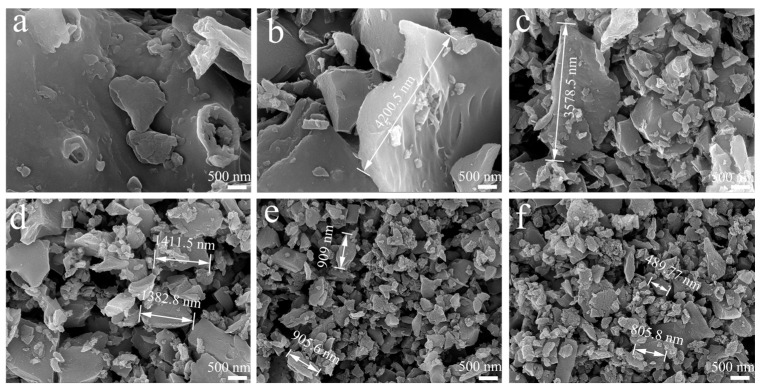
The SEM images of (**a**) the B-0, (**b**) B-1, (**c**) B-3, (**d**) B-6, (**e**) B-12, and (**f**) B-18.

Figure 3a–f presents the morphological evolution of filler particles under microscopic observation, clearly demonstrating the progressive reduction in WSB particle size with ball-milling treatment. The unprocessed WSB particles exhibited large dimensions with irregular geometries, rough surfaces, and limited porosity (Figure 3a). With increasing ball-milling duration, a gradual reduction in carbon particle dimensions accompanied by improved size uniformity became evident, consistent with the particle size analysis results. Concurrently, the particle surfaces transitioned to smoother textures with progressive elimination of porous structures (Figure 3b–f). These SEM observations further confirm that ball milling effectively achieves uniform particle size reduction in carbon materials through mechanical processing.

**Figure 4:**
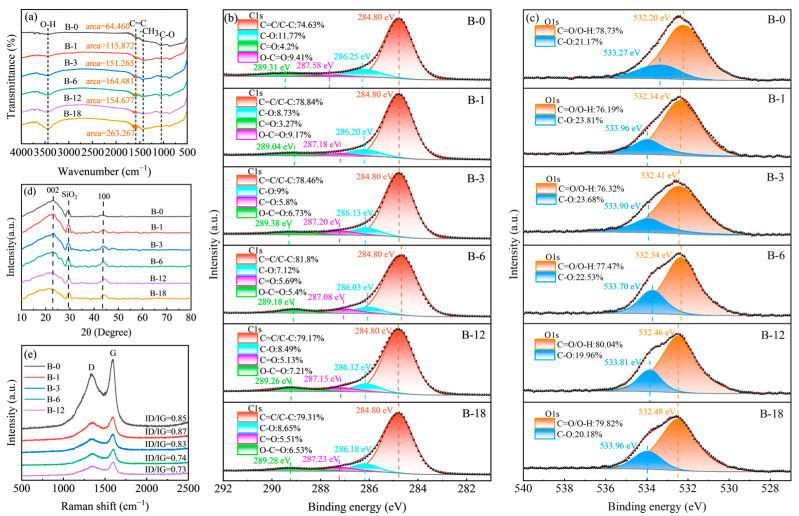
(**a**) FTIR results of the ball-milled products; (**b**) XPS C1s spectra of the ball-milled products; (**c**) XPS O1s spectra of the ball-milled products; (**d**) XRD results of the ball-milled products; and (**e**) Raman spectra of the ball-milled products.

The surface properties of carbon materials significantly influence their reinforcement performance. To investigate the surface chemical properties of WSB during the ball-milling process, FTIR analysis was first performed on the samples (Figure 4a). In the FTIR spectra, the -OH stretching vibration peak at 3441 cm^−1^ of ball-milled WSB was observed to significantly intensify with prolonged milling time [28]. The absorption peak at 1590 cm^−1^, attributed to the aromatic C=C stretching vibration, exhibited a progressive increase in integrated peak area. This phenomenon may be associated with enhanced graphitization degree of WSB. According to the literature, biochar containing graphitic domains has been reported to enhance interfacial compatibility with rubber. The graphitic structure facilitates the slippage of rubber molecular chains along the graphitic domains, whereas defects at the edge regions of these domains serve as active sites. These defects can adsorb rubber molecules either through physical interactions (van der Waals forces) or via cross-linking reactions with the rubber vulcanization system [24]. The absorption peak observed at 1439 cm^−1^ corresponds to the asymmetric angular vibration of -CH_3_ groups. Additionally, the characteristic peak at 1050 cm^−1^ is attributed to the stretching vibration of C-O bonds [29].

The XPS analysis demonstrated that the ball-milling process led to a significant increase in the surface oxygen content of the carbon material. The C1s spectra were deconvoluted into four characteristic peaks located at 284.8, 286.2, 287.2, and 289.3 eV, corresponding to C-C, C-O, C=O, and O-C=O functional groups, respectively. The O1s spectra exhibited two fitted peaks at 532.3 and 533.8 eV, assigned to C=O and C-O species [30], with oxygen predominantly existing in the C=O configuration (Figure 4b,c). The total oxygen content demonstrated a non-monotonic variation with ball-milling duration, initially decreasing to a minimum value of 7% at 3 h, then increasing to 9.32% at 6 h, before declining again to 7.64% after 18 h. This phenomenon can be attributed to the initial reduction in O-C=O groups with lower thermal decomposition temperatures [31], followed by oxygen content elevation through mechanochemical introduction of oxygen-containing functional groups. Ultimately, prolonged mechanical shearing and localized thermal effects during extended ball milling promoted gradual decomposition of oxygen-containing moieties, leading to the final oxygen content reduction (Table 1) [32]. The XPS analysis demonstrated that an appropriate ball-milling duration enhances the oxygen content of WSB, thereby increasing the density of active sites on the carbon material surface. This augmentation of active sites facilitates enhanced interfacial interactions between WSB and rubber molecular chains, which consequently improves the mechanical properties of the rubber composite.

The preceding FTIR results have demonstrated the potential of ball milling to enhance the graphitization degree of WSB. Furthermore, literature evidence indicates that ball milling effectively disrupts C=C bonds, inducing fragmentation of initial WSB particles while simultaneously generating a substantial number of highly reactive lattice defects, dangling bonds, and carbon radicals. Under continuous mechanical energy input, carbon atoms at these newly formed reactive sites readily rearrange into low-energy graphite lattices, leading to defect healing and pore elimination, thereby significantly enhancing the graphitization degree. This hypothesis is corroborated by XRD and Raman spectroscopy analyses. In the XRD patterns (Figure 4d), the (002) diffraction peak and (100) characteristic peak align with the stacking thickness and lateral dimensions of graphene sheets in graphite [33]. It can be observed that the (002) peak appears weak and broad, which is characteristic of disordered carbon, indicating its highly disordered structure. However, with increasing ball-milling time, an enhancement in the peak intensity at (100) has been observed, suggesting that the carbon structure is becoming progressively more ordered. To further validate the ball milling-induced graphitization of biochar, Raman spectroscopy was conducted. Raman results demonstrate a gradual decrease in ID/IG values with prolonged ball-milling duration, reaching stabilization without significant decrease after 6 h (Figure 4e). These results demonstrate that ball milling effectively repaired the structural defects of biochar and enhanced its graphitization degree. The analytical findings collectively reveal that the mechanical forces generated during ball milling disrupted the structural integrity of biochar, resulting in diminished particle dimensions (Figure 2b,c). The mechanical forces of ball milling induced the generation of oxygen and carbon radicals through defect-rich states on the biochar surface (Table 1). These newly formed active sites underwent structural rearrangement to establish graphite-like crystalline lattice features (Figure 4d), ultimately leading to the distinct formation of graphitic structures in the biochar matrix (Figure 4e).

### 2.2. Properties of Rubber Composites

**Figure 5:**
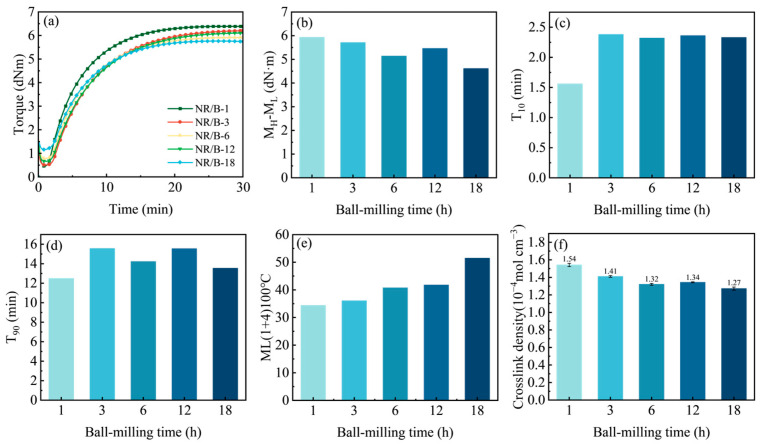
(**a**) Vulcanization curve of rubber compounds (NR/B-1: natural rubber composites were filled using WSB with ball-milling time of 1 h, and so on for NR/B-3, NR/B-6, NR/B-12 and NR/B-18); (**b**) M_H_-M_L_ of rubber compounds; (**c**) T_10_ of rubber compounds; (**d**) T_90_ of rubber compounds; (**e**) Mooney viscosity of rubber compounds; (**f**) crosslink density of rubber compounds.

Figure 5a–e demonstrate the effect of WSB with different ball-milling durations on the vulcanization properties of compounded rubber materials. The results demonstrated that the minimum torque (M_L_) of NR/B-1 measured merely 0.46 dNm, while NR/B-18 exhibited the maximum M_L_ value of 1.16 dNm. The M_L_ values followed this descending order: NR/B-18 > NR/B-6 > NR/B-12 > NR/B-3 > NR/B-1. Higher M_L_ values indicate lower fluidity of rubber compounds during the initial vulcanization stage, which can be attributed to the ball-milling process enhancing the specific surface area of WSB. This increased surface area promotes greater physical cross-linking between rubber molecular chains and WSB particles, thereby elevating the compound’s initial flow resistance as particle size decreases (Figure 5a). For natural rubber compounds, the (M_H_-M_L_) values displayed this descending sequence: NR/B-1 > NR/B-3 > NR/B-12 > NR/B-6 > NR/B-18. The composite materials reinforced with smaller particle-sized biochar exhibit a reduced chemical crosslinking ratio, while the analysis indicates an elevated proportion of physical entanglement. This phenomenon is attributed to the decreased particle dimensions of the carbon black filler (Figure 5b). The scorch time (T_10_) and optimal curing time (T_90_) exhibited an increase during the initial 3 h ball-milling period, but showed no significant changes beyond 3 h (Figure 5c,d). This phenomenon may be attributed to the larger particle size of WSB after short-term ball milling, which tends to agglomerate within the rubber matrix. Such agglomeration facilitates accelerated local heat transfer that promotes the vulcanization reaction. In contrast, prolonged ball milling enables more uniformly dispersed WSB particles in the rubber matrix while enhancing interfacial interactions between WSB and the rubber matrix. The Mooney viscosity exhibited a progressive increase with prolonged ball milling duration of WSB, indicating reduced fluidity and elevated relative molecular weight in the composite material. This phenomenon can be attributed to the ball-milling process enhancing the specific surface area of WSB, thereby increasing the effective surface area of carbon black per unit volume of rubber (Figure 5e) [34]. Concurrently, structural rearrangement of the biochar occurred post-milling, accompanied by an enhanced graphitization degree. This structural modification may facilitate conjugate adsorption with unsaturated double bonds present in natural rubber. The resultant interfacial interactions consequently elevated the Mooney viscosity while simultaneously improving the performance characteristics of the natural rubber composite.

The crosslink density data exhibited a consistent trend with the variations in M_H_ and M_H_-M_L_ (Figure 5f). The specific crosslink density values followed this descending order: NR/B-1 > NR/B-3 > NR/B-12 > NR/B-6 > NR/B-18. This phenomenon can be primarily attributed to the larger particle size and rougher surface of WSB with shorter ball-milling duration, which restricts the mobility of rubber molecular chains. Concurrently, localized agglomeration of WSB may lead to concentrated distribution of curing agents, thereby inducing regionally elevated crosslink density.

**Figure 6:**
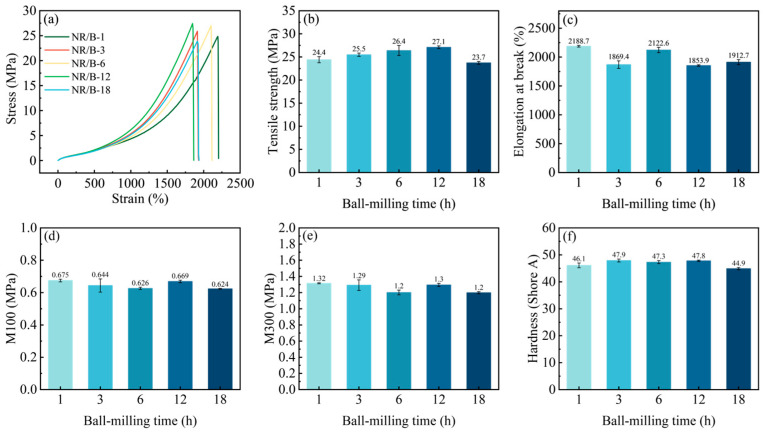
Mechanical properties of vulcanizates filled with different WSB: (**a**) stress-strain, (**b**) tensile strength, (**c**) elongation at break, (**d**) M_100_, (**e**) M_300_, and (**f**) hardness shore A of composites.

Figure 6a–f present the physicomechanical properties of WSB-filled NR vulcanizates under varying ball-milling times. As shown in Figure 6a,b, the tensile strength demonstrated an initial increase followed by a subsequent decrease with prolonged ball-milling duration. The NR/B-12 composite exhibited the maximum tensile strength of 27.1 MPa. This phenomenon can be attributed to the optimized particle size and exposed active functional groups of WSB after appropriate ball-milling treatment. The reduced particle dimensions facilitated uniform dispersion within the rubber matrix, while the enhanced graphitization degree of WSB improved interfacial interactions between the biochar filler and rubber molecular chains. These synergistic effects promoted the formation of bound rubber through strengthened physical adsorption and chemical compatibility, thereby effectively restricting molecular chain mobility and ultimately enhancing the mechanical performance of the composite [12]. The elongation at break of the composite materials exhibited minimal variation across different ball-milling durations (Figure 6c). Both the 100% modulus (M_100_) and 300% modulus (M_300_) of the composites followed a trend similar to that of crosslinking density, while the hardness values remained largely unchanged (Figure 6d–f). This further confirms the cross-linking effects of WSB in the rubber matrix under different ball-milling durations.

**Figure 7:**
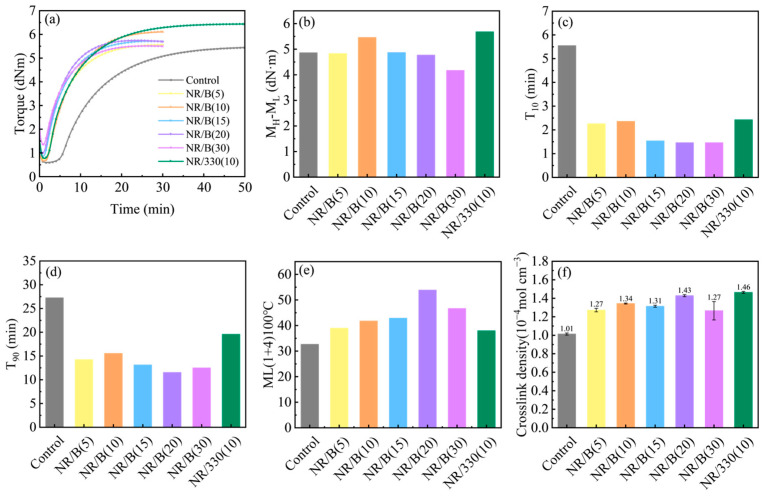
(**a**) Vulcanization curve of rubber compounds (NR/B(5): natural rubber composites contain 5 phr of WSB, and so on for NR/B(10), NR/B(15), NR/B(20), NR/B(30), and NR/330(10)); (**b**) M_H_-M_L_ of rubber compounds; (**c**) T_10_ of rubber compounds; (**d**) T_90_ of rubber compounds; (**e**) Mooney viscosity of rubber compounds; (**f**) crosslink density of rubber compounds.

**Figure 8:**
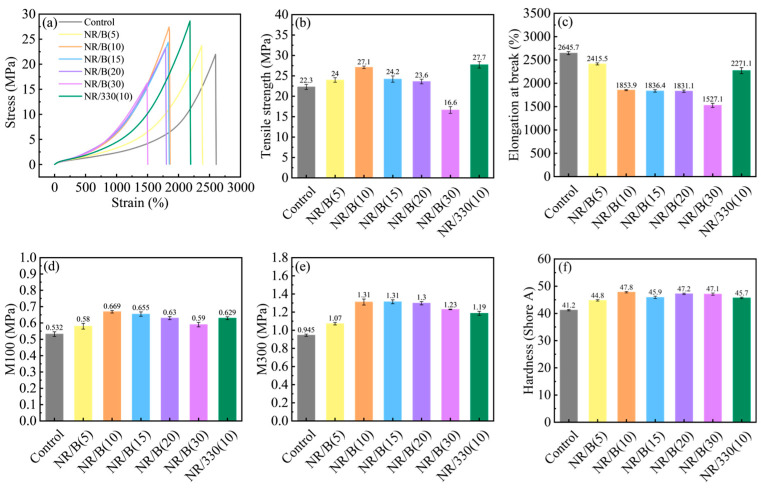
Mechanical properties of vulcanizates with different WSB additions: (**a**) stress-strain; (**b**) tensile strength; (**c**) elongation at break; (**d**) M_100_; (**e**) M_300_; and (**f**) hardness shore A of composites.

Figure 7a–e illustrate the vulcanization characteristics of rubber material without reinforcing agents, natural rubber composites containing varying contents of WSB, and N330-filled natural rubber material. Compared with the control rubber composite without biochar addition, the rubber material incorporated with WSB exhibits increased M_L_ (Figure 7a). This phenomenon arises because the increased filler loading of WSB particles restricts the slippage of rubber molecular chains, requiring greater external force to overcome internal resistance, thereby enhancing material rigidity. Both M_H_ and its difference from M_L_ (M_H_-M_L_) demonstrate an initial increase followed by a decreasing trend with elevated WSB content. The NR/B(10) composite achieves maximum M_H_ and M_H_-M_L_ values of 6.12 dNm and 5.46 dNm, respectively. However, these parameters significantly decrease when WSB content reaches 30 phr, attributable to excessive alkaline WSB adsorbing the acidic accelerator DM, which reduces available DM for crosslinking reactions and consequently diminishes chemical crosslink network formation [35]. The NR/B(10) composite exhibits the highest chemical crosslinking density, corresponding to its maximum tensile strength, a finding consistent with the mechanical property analysis of rubber composites (Figure 8a). Figure 7f presents the crosslinking density of rubber materials. The results demonstrate that the incorporation of WSB enhances the crosslinking density of rubber composites, though the overall difference remains relatively minor. This phenomenon can be attributed to both the enhanced rubber-WSB interfacial interactions and the formation of WSB-derived network structures. Notably, the composite containing 10 phr of biochar exhibits prolonged scorch time, indicating enhanced processing safety during manufacturing.

Figure 8a–f presents the physical–mechanical properties of unreinforced rubber material, natural rubber composites containing different WSB contents, and N330-filled natural rubber material. The results demonstrate that the tensile strength of vulcanizates was enhanced with filler addition. When incorporating 10 phr WSB, the NR/B(10) composite achieved a maximum tensile strength of 27.1 MPa, while its elongation at break gradually decreased (Figure 8a–c). This enhancement mechanism originates from the increased carbon particle content and volume fraction, which restricts the movement of rubber molecular chains. However, at 30 phr WSB loading, the tensile strength of NR/WSB composite declined to 16.6 MPa. This deterioration can be attributed to the excessive WSB content exceeding its dispersion threshold during mixing. Agglomeration of WSB particles reduces the proportion of effective biochar–rubber network structures and simultaneously hinders the orientation alignment of rubber macromolecular chains. These structural defects induce stress concentration under external forces, ultimately leading to performance degradation of the vulcanizates [36]. With the increase of WSB content, the M_100_ and M_300_ values of the vulcanized rubber exhibited a trend of initial increase followed by decrease, which was similar to that observed in tensile strength variation. Meanwhile, the Shore A hardness demonstrated an enhancement. When the WSB content reached 10 parts, the M_100_ value measured 0.669 MPa, the M_300_ value attained 1.31 MPa, and the hardness increased to 47.8 Shore A, as shown in Figure 8d–f.

In the experiment, commercial N330 carbon black was used to reinforce NR as a comparative study against modified biochar (WSB) for NR reinforcement. The M_H_ and M_H_-M_L_ values of NR/B(10) showed slight decreases compared to NR/330(10) (Figure 7a,b). This phenomenon is attributed to the smaller particle size and larger specific surface area of CB, which facilitates stronger adsorption of rubber molecular chains through van der Waals forces, thereby more effectively restricting molecular mobility. The Mooney viscosity demonstrated an increasing trend with filler loading (Figure 7e), resulting from the hydrodynamic effects of WSB in the rubber matrix [34]. Notably, rubber compounds containing WSB exhibited significantly reduced scorch time and vulcanization time (Figure 7c,d), indicating that WSB incorporation effectively enhanced industrial processing efficiency. This acceleration mechanism may stem from two factors: (1) improved thermal conductivity efficiency within the rubber mixture through WSB addition, facilitating faster heat transfer to promote vulcanization reactions; (2) activation of vulcanization by reactive functional groups present in WSB [24]. In contrast, N330 particles exhibited delayed vulcanization rates due to their high specific surface area adsorbing vulcanizing agents. The vulcanization parameters indicate that an appropriate dosage of WSB exerts a reinforcing effect on natural rubber. WSB enhances the crosslink density of the rubber system (Figure 7f), while simultaneously improving production efficiency in rubber manufacturing processes. Compared to the control rubber without reinforcing agents, the WSB-vulcanized rubber with 10 phr additive demonstrated a 22% improvement in tensile strength (Figure 8b). The WSB-filled vulcanizate achieved comparable tensile strength to the N330-containing counterpart (10 phr), reaching 98% of its performance (Figure 8b). The NR/WSB vulcanizates prepared in this study have achieved a significant enhancement in mechanical properties, which has rarely been reported in previous research. Crosslink density analysis revealed that NR/B(10) exhibited lower values than NR/330(10) (Figure 7f). This disparity mainly originates from N330’s smaller particle size and higher specific surface area. While a performance gap persists between biochar and commercial carbon black, our results confirm that ball milling effectively optimizes WSB through three key modifications: refined particle size distribution, increased active sites, and developed graphitic structures. The incorporation of these structural modifications enables WSB to effectively reinforce natural rubber composites.

**Figure 9:**
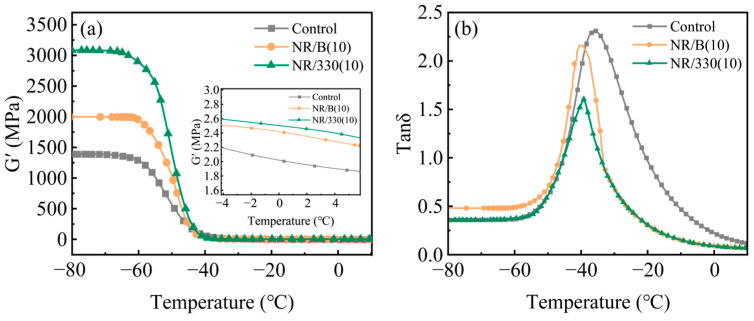
The temperature dependence of (**a**) G′, (**b**) tan δ of the control, NR/B(10), and NR/330(10) vulcanizates.

Dynamic Thermomechanical Analysis (DMA) can be employed to elucidate the microstructure and interfacial interactions in composite materials. A comparative analysis was conducted on three material systems: the unfilled natural rubber matrix, the optimum-performance biochar-reinforced natural rubber composite, and the commercial carbon black-filled natural rubber composite system. Figure 9a,b presents the temperature dependence of storage modulus (G′) and loss factor (tan δ) for rubber composite materials and rubber composites without reinforcing agents [37]. The NR/B(10) vulcanizate exhibits higher G′ than unreinforced rubber but lower than NR/330(10) vulcanizate, indicating weaker filler network structure and inferior filler-rubber interactions in NR/B(10) compared to NR/330(10). Furthermore, the diminished filler network structure in NR/B(10) generates enhanced internal friction, resulting in a higher tan δ peak than N330, while its weaker filler–rubber interactions lead to lower Tg. The tan δ values of NR/B(10) composites surpass those of N330 but remain below unreinforced rubber, demonstrating that biochar-reinforced composites exhibit superior elastic recovery and reduced hysteresis loss. However, the reinforcement efficiency still lags behind commercial carbon black. Nevertheless, WSB effectively enhances the mechanical properties of natural rubber, achieving comparable performance to commercial CB at lower cost.

## 3. Discussion

In summary, we successfully prepared biochar derived from walnut shells and fabricated biomass carbon black through physical ball milling for natural rubber reinforcement. The effects of ball-milling duration on the physicochemical structure of biomass carbon black were systematically investigated through gradient experiments, with subsequent exploration of its reinforcing effects on natural rubber composites. This study aimed to develop an environmentally friendly biochar as a reinforcing material for natural rubber. Experimental results demonstrated that ball milling effectively reduced particle size, enhanced surface reactivity, and modified the structural characteristics of WSB. Raman spectroscopy analysis confirmed that ball milling significantly improved the graphitization degree of biochar. Experimental data on vulcanization and mechanical properties demonstrate that the NR composite filled with 12 h ball-milled biochar achieves a tensile strength of 27.1 MPa. The NR/WSB composite exhibits superior vulcanization characteristics compared to unfilled rubber materials. At 10 phr WSB loading, the vulcanizate displays optimal mechanical performance, showing 22% enhancement over the biochar-free composite and achieving 98% of the reinforcing efficiency observed in commercial carbon black N330-filled composites. Dynamic thermomechanical analysis reveals that biochar incorporation effectively reduces hysteresis loss in natural rubber, thereby improving elastic recovery. The underlying mechanism responsible for the performance improvement has not been conclusively determined, as it may stem from either the physical characteristics of biochar or its elevated graphitization degree, warranting additional systematic studies. These findings contribute to the development of renewable carbon black alternatives, positioning biochar as a potential reinforcing filler for rubber industry applications.

## 4. Materials and Methods

### 4.1. Experimental Materials

The natural latex was purchased from Tianbang Latex Co., Ltd. (Mengla, China). Carbon black (CB, N330) was obtained from Yunnan Yunwei Feihu Chemical Co., Ltd. (Qujing, China). Acetic acid was procured from Shanghai Titan Technology Co., Ltd. (Shanghai, China). Other chemical reagents, including zinc oxide, stearic acid, accelerator DM, and sulfur, were commercially available industrial-grade materials. Walnut shells were collected from local sources in Yunnan Province, China.

### 4.2. Preparation of Ball-Milled Walnut Shell Carbon

The preparation process was conducted as follows: Firstly, walnut shells were thoroughly cleaned and dried at 80 °C to remove moisture. Subsequently, the dried shells were pulverized to achieve uniform particle size through 100-mesh sieving. Subsequently, the obtained walnut shell powder was placed in a tubular furnace. Under a nitrogen atmosphere with a flow rate of 100 cm^3^/min, the material was heated to 600 °C at a heating rate of 10 °C/min and maintained for 2 h for carbonization. For the ball-milling procedure, the WSB was mixed with deionized water at a mass ratio of 1:3 (WSB:H_2_O) in a 500 mL milling container. The ball-milling process was performed at 500 r/min rotational speed with duration variations of 0, 1, 3, 6, 12, and 18 h. The resultant samples were systematically designated as B-T, where T represents the ball-milling time in hours (e.g., B-1 denotes the sample processed for 1 h). Detailed parameters of ball-milled samples are summarized in Table 2.

### 4.3. Preparation of NR/WSB Vulcanizates

The walnut shell biochar dispersion was initially introduced into the 20% natural rubber latex under gradual addition, followed by ultrasonication-assisted stirring for 10 min. Subsequently, the mixture underwent sequential processing including flocculation, shearing, washing, and oven-drying at 50 °C, ultimately yielding the NR/WSB raw rubber. The prepared raw rubber was uniformly blended with vulcanizing agents, accelerators, and other additives using a two-roll mill. The mixture was then hot-pressed at 150 °C for optimal curing time to complete the vulcanization process. The specific rubber formulations are presented in Table 3 and Table 4.

### 4.4. Methods

The particle size distribution of WSB filler was determined by laser diffraction particle size analyzer (Malvern Mastersizer 2000, Malvern Panalytical Ltd, Malvern, United Kingdom). Surface characterization was performed using Fourier transform infrared spectroscopy (FTIR, Bruker Vertex 70, Bruker Optik GmbH, Ettlingen, Germany) and X-ray photoelectron spectroscopy (XPS, Thermo Scientific ESCALAB 250Xi, Thermo Fisher Scientific Inc., Waltham, MA, USA). The morphology of carbon particles was examined through scanning electron microscopy (SEM, Hitachi Regulus 8100, Hitachi High-Tech Corporation, Tokyo, Japan). The structural properties of carbon materials were characterized by X-ray diffraction (XRD, Rigaku Ultima IV, Rigaku Corporation, Tokyo, Japan) and Raman spectroscopy (Horiba LabRam HR Evolution, HORIBA, Ltd., Kyoto, Japan).

The vulcanization parameters were determined using an MDR-2000E rotorless rheometer with the test temperature maintained at 150 °C. The Mooney viscosity was measured on a MV2-2000 computer-controlled Mooney viscometer in compliance. The mechanical properties of vulcanized rubber were evaluated through tensile testing performed on a ZQ-990LB electric tensile testing machine [23]. Shore hardness measurements were conducted using an LXD-A digital Shore durometer. Dynamic thermal properties of NR/WSB vulcanized rubber were characterized by a Q800 dynamic mechanical analyzer (TA Instruments Ltd New Castle, DE, United States) in tension mode, employing a test frequency of 10 Hz across a temperature range from −80 °C to 10 °C, with a controlled heating rate of 3 °C/min.

The vulcanized rubber sample was immersed in toluene for 72 h until equilibrium swelling was achieved. The swollen sample was promptly removed from the solvent, with its surface toluene thoroughly blotted dry using filter paper, and subsequently weighed. The swollen specimen was then transferred to a vacuum oven and dried at 50 °C until reaching constant mass, followed by a final weighing measurement. The volume fraction of rubber was calculated using Equation (1), which was subsequently employed in Equation (2) to determine the crosslink density of the material.

(1) $$v_{1}=\frac{m_{3}∕\rho_{1}}{m_{3}∕\rho_{1}+\left(m_{2}-m_{1}\right)∕\rho_{2}}$$

In the equation, *V*_1_ represents the volume fraction of rubber, where *m*_1_ denotes the mass of the unswollen sample, *m*_2_ corresponds to the mass of the swollen sample after solvent absorption, and *m*_3_ indicates the mass of the dried sample after deswelling. The parameters *ρ*_1_ and *ρ*_2_ refer to the densities of natural rubber (0.920 g/cm^3^) and toluene (0.865 g/cm^3^), respectively. This relationship adheres to the thermodynamic principles of polymer–solvent interactions during the swelling equilibrium process.

(2) $$\rho =-\frac{\ln\left(1-v_{1}\right)+V_{1}+av_{1}^{2}}{v_{2}\left(\sqrt[{3}]{v_{1}}-0.5v_{1}\right)}$$

where *ρ* is the crosslink density, a represents the interaction parameter between rubber and toluene (0.393), and *V*_2_ denotes the molar volume of toluene (106.5 mL/mol).

## Figures and Tables

**Table 1:** Elemental composition of different WSB.

Samples	Element Composition (At%)	
	C1s	O1s
B-0	90.26	8.34
B-1	90.55	8.27
B-3	92.17	7
B-6	89.36	9.32
B-12	90.05	8.97
B-18	91.21	7.64

**Table 2:** Ball milling parameters of WSB.

Samples	Milling Time (h)
B-0	0
B-1	1
B-3	3
B-6	6
B-12	12
B-18	18

**Table 3:** WSB-filled rubber composite formulations with different ball-milling times.

Component	Formula (1) (phr ^a^)	Formula (2) (phr)	Formula (3) (phr)	Formula (4) (phr)	Formula (5) (phr)
Natural Rubber	100	100	100	100	100
B-1	10	0	0	0	0
B-3	0	10	0	0	0
B-6	0	0	10	0	0
B-12	0	0	0	10	0
B-18	0	0	0	0	10
Zinc oxide	5	5	5	5	5
Stearic acid	3	3	3	3	3
Sulfur	2.5	2.5	2.5	2.5	2.5
Promoter DM	0.6	0.6	0.6	0.6	0.6

^a^ phr denotes the number of parts per hundred parts of rubber.

**Table 4:** Rubber compound formulations.

Component	Formula (1) (phr ^a^)	Formula (2) (phr)	Formula (3) (phr)	Formula (4) (phr)	Formula (5) (phr)	Formula (6) (phr)	Formula (7) (phr)
Natural Rubber	100	100	100	100	100	100	100
B-12	0	5	10	15	20	30	0
N330	0	0	0	0	0	0	10
Zinc oxide	5	5	5	5	5	5	5
Stearic acid	3	3	3	3	3	3	3
Sulfur	2.5	2.5	2.5	2.5	2.5	2.5	2.5
Promoter DM	0.6	0.6	0.6	0.6	0.6	0.6	0.6

^a^ phr denotes the number of parts per hundred parts of rubber.

## Data Availability

The data presented in this study are available on request from the corresponding author.

## References

[1] Al-Hartomy O.A., Al-Solamy F., Al-Ghamdi A., Dishovsky N., Ivanov M., Mihaylov M., El-Tantawy F. (2011). Influence of Carbon Black Structure and Specific Surface Area on the Mechanical and Dielectric Properties of Filled Rubber Composites. Int. J. Polym. Sci..

[2] Ismail H., Omar N.F., Othman N. (2011). Effect of carbon black loading on curing characteristics and mechanical properties of waste tyre dust/carbon black hybrid filler filled natural rubber compounds. J. Appl. Polym. Sci..

[3] Li Y., Zhu P., Zhang Q., Chen B., Zhu Z. (2019). Study on the Properties of Rubber with Different Contents of Carbon Black. IOP Conf. Ser. Mater. Sci. Eng..

[4] Kang H., Tang Y., Yao L., Yang F., Fang Q., Hui D. (2017). Fabrication of graphene/natural rubber nanocomposites with high dynamic properties through convenient mechanical mixing. Compos. Part B Eng..

[5] Savetlana S., Sukmana I., Saputra F.A. (2017). The effect of carbon black loading and structure on tensile property of natural rubber composite. IOP Conf. Ser. Mater. Sci. Eng..

[6] Abdelsalam A.A., Araby S., El-Sabbagh S.H., Abdelmoneim A., Hassan M.A. (2021). Effect of carbon black loading on mechanical and rheological properties of natural rubber/styrene-butadiene rubber/nitrile butadiene rubber blends. J. Thermoplast. Compos. Mater..

[7] Chang B.P., Gupta A., Muthuraj R., Mekonnen T.H. (2021). Bioresourced fillers for rubber composite sustainability: Current development and future opportunities. Green Chem..

[8] Cha J.S., Park S.H., Jung S.C., Ryu C., Jeon J.K., Shin M.C., Park Y.K. (2016). Production and utilization of biochar: A review. J. Ind. Eng. Chem..

[9] Greenough S., Dumont M.J., Prasher S. (2021). The physicochemical properties of biochar and its applicability as a filler in rubber composites: A review. Mater. Today Commun..

[10] Bélanger N., Prasher S., Dumont M.J. (2023). Tailoring biochar production for use as a reinforcing bio-based filler in rubber composites: A review. Polym. Plast. Technol. Mater..

[11] Jong L., Peterson S.C., Jackson M.A. (2014). Utilization of Porous Carbons Derived from Coconut Shell and Wood in Natural Rubber. J. Polym. Environ..

[12] Lay M., Rusli A., Abdullah M.K., Hamid Z.A.A., Shuib R.K. (2020). Converting dead leaf biomass into activated carbon as a potential replacement for carbon black filler in rubber composites. Compos. Part B Eng..

[13] Zainal Abidin Z., Mamauod S.N.L., Romli A.Z., Sarkawi S.S., Zainal N.H. (2023). Synergistic effect of partial replacement of carbon black by palm kernel shell biochar in carboxylated nitrile butadiene rubber composites. Polymers.

[14] Mago J., Negi A., Pant K.K., Fatima S. (2022). Development of natural rubber-bamboo biochar composites for vibration and noise control applications. J. Clean. Prod..

[15] Dai A., Wu Q., Xu C., Xiong J., Fan L., Ke L., Zeng Y., Cobb K., Ruan R., Wang Y. (2024). Walnut shell oil-bath torrefaction coupled with fast pyrolysis: Effect of torrefaction heating modes. Bioresource Technol..

[16] Zhou S., Wei Y., Li B., Wang H. (2019). Cleaner recycling of iron from waste copper slag by using walnut shell char as green reductant. J. Clean. Prod..

[17] Chundawat N.S., Parmar B.S., Deuri A.S., Vaidya D., Sepehr K.S., Chauhan N.P.S. (2020). Walnut shell ash as a sustainable material for compounding with bromobutyl rubber for tire inner liner applications. Polym. Compos..

[18] Bokobza L. (2018). Natural rubber nanocomposites: A review. Nanomaterials.

[19] Gusiatin M.Z., Rouhani A. (2023). Application of selected methods to modify pyrolyzed biochar for the immobilization of metals in soil: A review. Materials.

[20] Lyu H., Yu Z., Gao B., He F., Huang J., Tang J., Shen B. (2019). Ball-milled biochar for alternative carbon electrode. Environ. Sci. Pollut. Res..

[21] Yuan Y., Zhang N., Hu X. (2020). Effects of wet and dry ball milling on the physicochemical properties of sawdust derived-biochar. Instrum. Sci. Technol..

[22] Wang J., Tan Y., Yang H., Zhan L., Sun G., Luo L. (2023). On the adsorption characteristics and mechanism of methylene blue by ball mill modified biochar. Sci. Rep..

[23] Xue B., Wang X., Sui J., Xu D., Zhu Y., Liu X. (2019). A facile ball milling method to produce sustainable pyrolytic rice husk bio-filler for reinforcement of rubber mechanical property. Ind. Crops Prod..

[24] Jiang C., Bo J., Xiao X., Zhang S., Wang Z., Yan G., Wu Y., Wong C., He H. (2020). Converting waste lignin into nano-biochar as a renewable substitute of carbon black for reinforcing styrene-butadiene rubber. Waste Manag..

[25] Naghdi M., Taheran M., Brar S.K., Rouissi T., Verma M., Surampalli R.Y., Valero J.R. (2017). A green method for production of nanobiochar by ball milling- optimization and characterization. J. Clean. Prod..

[26] Jeon I.Y., Bae S.Y., Seo J.M., Baek J.B. (2015). Scalable Production of Edge-Functionalized Graphene Nanoplatelets via Mechanochemical Ball-Milling. Adv. Funct. Mater..

[27] Barrera C.S., Cornish K. (2017). Processing and mechanical properties of natural rubber/waste-derived nano filler composites compared to macro and micro filler composites. Ind. Crops Prod..

[28] Stankovich S., Piner R.D., Nguyen S.T., Ruoff R.S. (2006). Synthesis and exfoliation of isocyanate-treated graphene oxide nanoplatelets. Carbon.

[29] Zhu X., Li K., Zhang L., Wu X., Zhu X. (2018). Comparative study on the evolution of physicochemical characteristics of biochar produced from bio-oil distillation residue under different induction atmosphere. Energy Convers. Manag..

[30] Yuan T., He W., Yin G., Xu S. (2020). Comparison of bio-chars formation derived from fast and slow pyrolysis of walnut shell. Fuel.

[31] Yuan R., Dong Y., Hou R., Shang L., Zhang J., Zhang S., Chen X., Song H. (2023). Structural transformation of porous and disordered carbon during ball-milling. Chem. Eng. J..

[32] Chang D.W., Choi H.J., Jeon I.Y., Seo J.M., Dai L., Baek J.B. (2014). Solvent-free mechanochemical reduction of graphene oxide. Carbon.

[33] Li T., Zhang L., Dong L., Li C.Z. (2014). Effects of gasification atmosphere and temperature on char structural evolution during the gasification of Collie sub-bituminous coal. Fuel.

[34] Chollakup R., Suethao S., Suwanruji P., Boonyarit J., Smitthipong W. (2021). Mechanical properties and dissipation energy of carbon black/rubber composites. Compos. Adv. Mater..

[35] Fu W., Wang L., Huang J., Liu C., Peng W., Xiao H., Li S. (2019). Mechanical Properties and Mullins Effect in Natural Rubber Reinforced by Grafted Carbon Black. Adv. Polym. Technol..

[36] Rattanasom N., Prasertsri S., Ruangritnumchai T. (2009). Comparison of the mechanical properties at similar hardness level of natural rubber filled with various reinforcing-fillers. Polym. Test..

[37] Li Y., Han B., Wen S., Lu Y., Yang H., Zhang L., Liu L. (2014). Effect of the temperature on surface modification of silica and properties of modified silica filled rubber composites. Compos. Part A Appl. Sci. Manuf..

